# Genome-wide data discovery of genetic variations in an advanced rice mutant line, NMR191, with blast resistance and early maturity traits

**DOI:** 10.1016/j.dib.2026.113023

**Published:** 2026-06-24

**Authors:** Wan Dalila Wan Chik, Faiz Ahmad, Wan Iryani Wan Ismail, Kamil Mohd Abdul Rahman, Muhammad Fairuz Hisyam Rezuwan, Ainin Sofiya Kamaruzzaman, Mohammad Malek Faizal Azizi, Affrida Abu Hassan, Asma Aris, Muniroh Md Saad, Muhammad Adib Najmi Jaa’far, Siti Nurdiyana Yusof, Sobri Hussein

**Affiliations:** a Agrotechnology and Biosciences Division, Malaysian Nuclear Agency Bangi, 43000, Kajang, Selangor, Malaysia; b Faculty of Science and Marine Environment, Universiti Malaysia Terengganu, 21030 Kuala Nerus, Terengganu, Malaysia; c Faculty of Applied Sciences, Universiti Teknologi MARA, Cawangan Negeri Sembilan Kampus Kuala Pilah, 72000 Kuala Pilah, Negeri Sembilan, Malaysia; d Department of Biological Sciences and Biotechnology, Faculty of Science and Technology, Universiti Kebangsaan Malaysia, 43600 Bangi, Selangor, Malaysia

**Keywords:** Whole-genome resequencing, Genomics, Mutant, Breeding

## Abstract

Climate change poses increasing challenges to rice production and food security, particularly in tropical regions such as Malaysia. This article presents a whole-genome resequencing dataset of a Malaysian-developed advanced rice mutant line, NMR191, and its parental cultivar, Pongsu Seribu (PS2), generated through ion beam irradiation-induced mutagenesis. Sequencing was performed using the Illumina NovaSeq X platform at approximately 30 × coverage, producing high-quality paired-end reads. Clean reads were aligned to the *Oryza sativa Indica* reference genome (GCA_000004655), followed by genome-wide identification of single nucleotide polymorphisms (SNPs) and insertions/deletions (InDels). The dataset comprises 24.8 GB and 28.6 GB of clean sequencing data for NMR191 and PS2, respectively. Raw sequencing reads are publicly available in the NCBI Sequence Read Archive under accession numbers and SRR36593642. This dataset provides a valuable genomic resource for variant discovery, comparative genomics, and future studies in rice breeding and mutation research.

Specifications Table

**Table:** 

Subject	Biology
Specific subject area	Agricultural and Plant Sciences
Type of data	Genome sequence data
Data collection	Genomic data were generated using the Illumina NovaSeq X platform for whole-genome resequencing of the rice mutant line NMR191 and its parental cultivar, PS2. The Illumina protocol was followed to prepare the library using high-quality genomic DNA that was extracted from fresh leaf tissue. DNA fragmentation, adapter ligation, and PCR enrichment were used to create sequencing libraries. Paired-end sequencing was conducted to improve genome coverage and alignment accuracy. Raw reads in FASTQ format were subjected to initial quality assessment before downstream variant analysis.
Data source location	City/Town/Region: Kajang, Selangor Country: Malaysia Latitude and longitude (and GPS coordinates) for collected samples/data:] 2.9108° N, 101.7705° E
Data accessibility	The raw sequencing reads for the mutant line and its parental cultivar have been deposited in the National Center for Biotechnology Information (NCBI) database (https://www.ncbi.nlm.nih.gov/). The data accessibility information is as follows: Repository name: Whole Genome Sequencing and Genetic Analysis of An Advanced Rice Mutant Line (NMR191) with Blast Resistance and Early Maturity Traits Data identification number: SRR36593643 [1] Direct URL to data: https://trace.ncbi.nlm.nih.gov/Traces/?run=SRR36593643 Repository name: Whole Genome Sequencing and Genetic Analysis of a rice cultivar (PS2) with Blast Resistance Data identification number: SRR36593642 [2] Direct URL to data: https://trace.ncbi.nlm.nih.gov/Traces/?run=SRR36593642

## 1 Value of the Data

• Provides genome-wide SNP and InDel dataset for mutant and parental rice lines.
• Supports comparative genomics and downstream variant analysis.
• Public repository enables reuse and validation.
• Useful for future SNP marker development, comparative genomics, and genome-wide association studies (GWAS).
• Supports mutation breeding research.

## 2 Background

NMR191 is an advanced mutant rice (*Oryza sativa* L.*)* line developed from the traditional cultivar, PS2, through ion beam irradiation-induced mutagenesis. Mutation breeding using ion beam irradiation is an effective approach for generating heritable genetic variation in crop improvement programs [3]; however, the genome-wide effects of this mutagenesis method remain insufficiently characterized at nucleotide resolution. Advances in next-generation sequencing (NGS) technologies have enabled high-throughput whole-genome resequencing (WGS) for systematic detection of genomic variants, including SNPs and small insertions/deletions [4]. To facilitate genomic characterization of NMR191, paired-end sequencing libraries of both NMR191 and PS2 were generated and sequenced using the Illumina NovaSeq X platform. The resulting high-coverage sequencing data enable genome-wide comparison between the mutant line and its parental cultivar, allowing identification of DNA polymorphisms associated with ion beam irradiation-induced mutations. The raw sequencing reads have been deposited in a public repository to support transparency and reproducibility. This dataset serves as a useful resource for downstream analyses such as variant annotation, comparative genomics, functional genomics, and molecular marker development for rice breeding applications.

## 3 Data Description

**Fig. 1:**
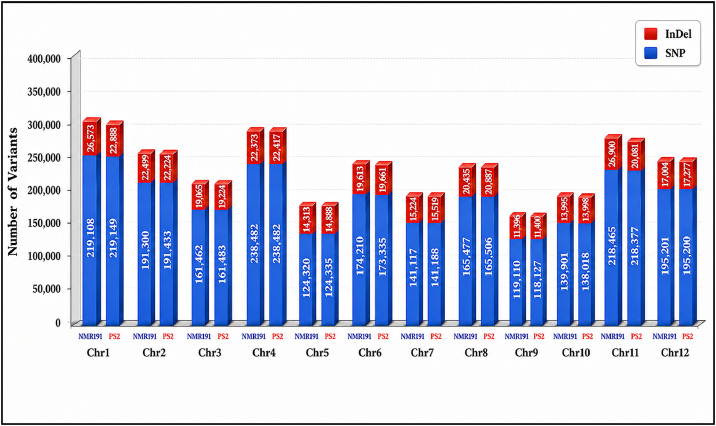
Distribution of single nucleotide polymorphisms (SNPs) and insertions/deletions (InDels) across the 12 chromosomes of the rice mutant line NMR191 and its parental cultivar, PS2. Blue bars represent SNP counts, while red bars indicate InDel counts identified through whole-genome resequencing against the *Oryza sativa indica* reference genome.

The dataset contains raw sequencing reads and associated metadata generated from whole-genome resequencing of the rice mutant line NMR191 and its parental cultivar, PS2. All sequencing data are publicly available in the National Center for Biotechnology Information (NCBI) repository under the corresponding accession numbers. The dataset includes FASTQ files containing paired-end sequencing reads as well as metadata describing the samples and sequencing information. The raw sequencing reads were generated using the Illumina NovaSeq X platform. Each sample consists of two FASTQ files representing paired-end reads (forward and reverse). These files contain nucleotide sequences and the repository includes metadata files describing sample information, sequencing platform, and data identifiers. Table 1 provides an overview of the dataset structure and file contents, while Fig. 1 summarizes the distribution of SNPs and InDels in NMR191 and PS2 relative to the reference genome. Most detected variants represent polymorphisms relative to the *Oryza sativa indica* reference genome shared by both NMR191 and PS2, while only a subset may correspond to irradiation-induced mutations.

**Table 1:** Description of dataset files and structure.

Genotype	Characteristics	Total raw nucleotides (bp)	Total clean nucleotides (bp)	Effective (%)	Q30 (%)	GC (%)	NCBI SRA accessions number
NMR191	Early maturity, high yield, blast resistant	25,268,466,900	24,820,597,800	98.55	96.16	43.36	SRR36593643
PS2	Blast-resistant, High-yield	29,115,420,300	28,550,580,300	98.06	96.44	43.70	SRR36593642

## 4 Experimental Design, Materials and Methods

### 4.1 Plant materials and DNA extraction

Leaf tissues were collected from NMR191 and PS2. A plant genomic DNA extraction protocol was used to extract the genomic DNA for high-throughput sequencing [5]. The quality and integrity of the extracted DNA were assessed using agarose gel electrophoresis to confirm high molecular weight DNA. DNA purity and concentration were measured using spectrophotometric and fluorometric quantification methods. DNA samples meeting the required quality standards were used to prepare sequencing libraries and whole-genome resequencing.

### 4.2 Whole-genome resequencing and data preprocessing

Whole-genome resequencing was performed using the Illumina NovaSeq X platform. Sequencing libraries were prepared and loaded onto the flow cell according to the manufacturer’s instructions [5]. Paired-end sequencing (2 × 150 bp) was conducted, enabling both ends of each DNA fragment to be sequenced [6]. Raw image data generated during sequencing were processed using the Illumina data processing pipeline to convert fluorescence signals into nucleotide base calls. The average sequencing depth was approximately 30 × for both samples. The resulting sequence reads were generated in FASTQ format, containing nucleotide sequences and corresponding base quality scores. Raw sequencing reads were subjected to quality control using Fastp (v0.20.0) with parameters -g -q 5 -u 50 -n 15 -l 150, which removed adapter sequences, low-quality reads, and reads containing ambiguous bases [6]. Quality evaluation included assessment of read length distribution, base quality scores, GC content, and sequencing yield. The resulting high-quality reads were retained for downstream analysis.

### 4.3 Genome alignment and variant identification

Filtered sequencing reads were aligned to the *Oryza sativa* reference genome (GCA_000004655) using BWA-MEM (v0.7.17-r1188) [7] with parameters mem -t 4 -k 32 -M. The alignment process mapped sequencing reads to their corresponding genomic positions in the reference genome. The resulting SAM files were converted to BAM format, sorted using SAMtools (v1.13) [8] with parameters sort -@ 6 -m 2 G, and indexed. BAM files from the same sample were merged using Picard (v1.111) with VALIDATION_STRINGENCY=SILENT. Variant detection was performed using SAMtools mpileup (v1.3.1) with parameters -m 2 -F 0.002 -q 1 -C 50 -t SP -t DP -t AD, enabling identification and preliminary filtering of SNPs and InDels [9]. Additional filtering criteria were applied, including minimum variant frequency ≥90%, minimum read depth ≥10, and base quality score ≥30, to retain high-confidence variants [10]. Functional annotation of detected variants was conducted using ANNOVAR (version 2015Dec14), allowing classification of variants into genomic regions such as coding sequences, introns, and intergenic regions [11].

#### 4.4 Data availability

All raw sequencing reads generated in this study were deposited in the National Center for Biotechnology Information (NCBI) repository. The dataset includes paired-end FASTQ files for both NMR191 and PS2 [1,2]. Accession numbers associated with the BioProject, BioSample, and Sequence Read Archive (SRA) entries are provided in the data accessibility section of this article.

**Fig. 2:**
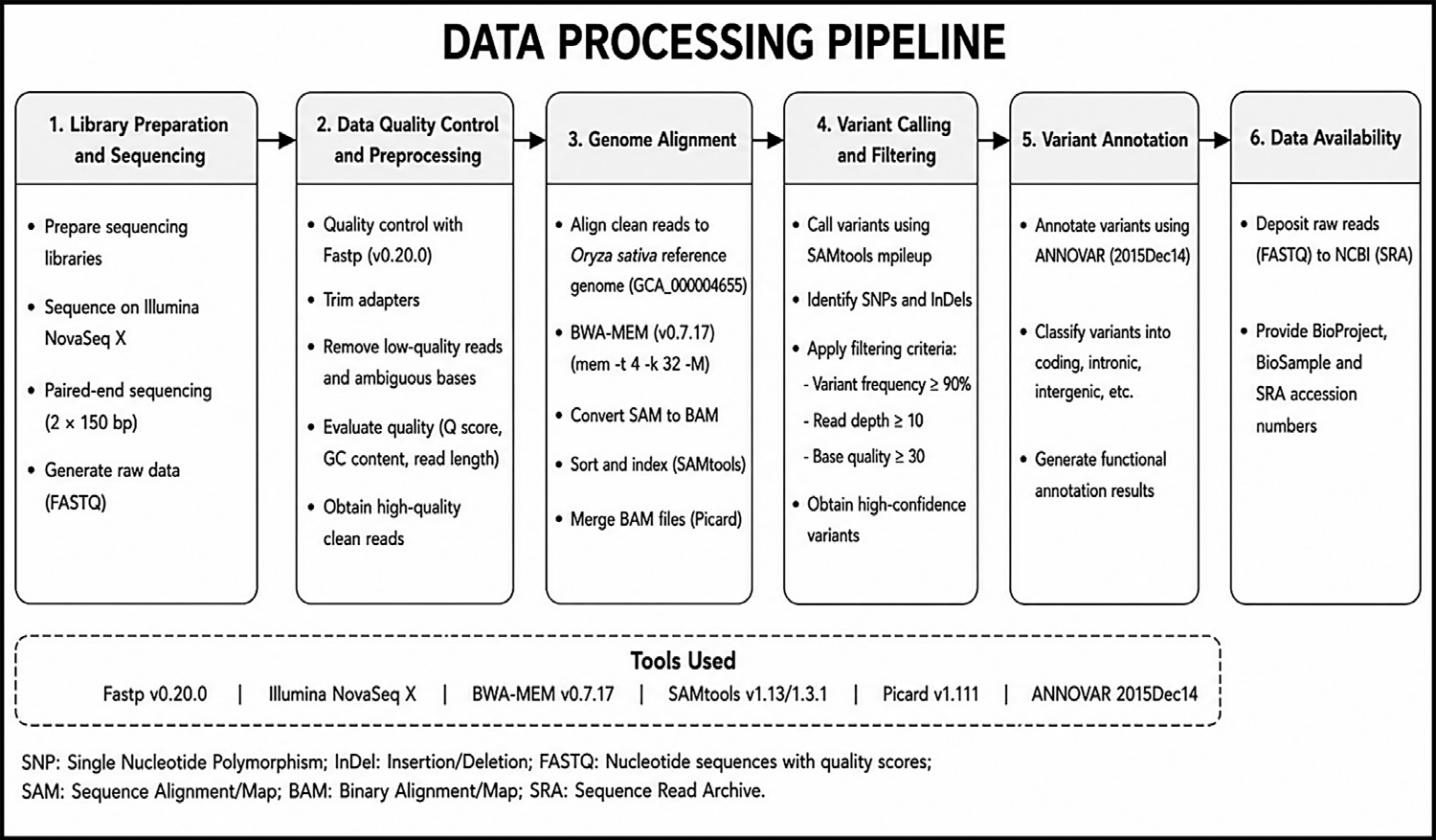
Data processing pipeline for whole-genome resequencing and variant analysis of the rice mutant line NMR191 and its parental cultivar PS2. The workflow includes sequencing library preparation, quality control and preprocessing of raw reads, genome alignment to the Oryza sativa reference genome, variant calling and filtering, functional annotation of SNPs and InDels, and public data deposition in the NCBI Sequence Read Archive (SRA).

The overall workflow for whole-genome resequencing, data preprocessing, genome alignment, variant identification, annotation, and data deposition is summarized in Fig. 2.

## Limitations

This dataset does not include experimental validation of identified variants and is intended as a resource for downstream validation and comparative genomic analyses.

## Ethics Statement

The authors have read and followed the ethical requirements for publication in Data in Brief. This study does not involve human participants, animal experiments, or data collected from social media platforms. The research was conducted using plant materials *(Oryza sativa)* in accordance with institutional and national guidelines.

## CRediT authorship contribution statement

**Wan Dalila Wan Chik:** Conceptualization, Investigation, Methodology, Data curation, Writing – original draft, Writing – review & editing. **Faiz Ahmad:** Investigation, Validation, Writing – review & editing. **Wan Iryani Wan Ismail:** Investigation, Methodology, Data curation, Writing – original draft, Writing – review & editing. **Kamil Mohd Abdul Rahman:** Investigation, Methodology, Data curation. **Muhammad Fairuz Hisyam Rezuwan:** Investigation, Methodology, Data curation. **Ainin Sofiya Kamaruzzaman:** Investigation, Methodology, Data curation. **Mohammad Malek Faizal Azizi:** Investigation, Validation, Writing – review & editing. **Affrida Abu Hassan:** Validation, Writing – review & editing. **Asma Aris:** Validation, Writing – review & editing. **Muniroh Md Saad:** Validation, Writing – review & editing. **Muhammad Adib Najmi Jaa’far:** Investigation. **Siti Nurdiyana Yusof:** Investigation. **Sobri Hussein:** Supervision, Validation, Writing – review & editing, Funding acquisition.

## Data Availability

NCBIWhole Genome Sequencing and Genetic Analysis of a rice cultivar (PS2) with Blast Resistance (Original data)
